# Immobilized TiO_2_ on glass spheres applied to heterogeneous photocatalysis: photoactivity, leaching and regeneration process

**DOI:** 10.7717/peerj.4464

**Published:** 2018-03-06

**Authors:** Deivisson Lopes Cunha, Alexei Kuznetsov, Carlos Alberto Achete, Antonio Eduardo da Hora Machado, Marcia Marques

**Affiliations:** 1 Department of Sanitary and Environmental Engineering, Universidade do Estado do Rio de Janeiro, Rio de Janeiro, Brazil; 2 Divisão de Metrologia de Materiais-DIMAT, Instituto Nacional de Metrologia, Qualidade e Tecnologia-INMETRO, Duque de Caxias, Rio de Janeiro, Brazil; 3 Laboratory of Photochemistry and Materials Science, Institute of Chemistry, Universidade Federal de Uberlândia, Uberlândia, Minas Gerais, Brazil

**Keywords:** Immobilized TiO_2_, Heterogeneous photocatalysis, Regeneration process, Dye degradation

## Abstract

Heterogeneous photocatalysis using titanium dioxide as catalyst is an attractive advanced oxidation process due to its high chemical stability, good performance and low cost. When immobilized in a supporting material, additional benefits are achieved in the treatment. The purpose of this study was to develop a simple protocol for impregnation of TiO_2_-P25 on borosilicate glass spheres and evaluate its efficiency in the photocatalytic degradation using an oxidizable substrate (methylene blue), in a Compound Parabolic Concentrator (CPC) reactor. The assays were conducted at lab-scale using radiation, which simulated the solar spectrum. TiO_2_ leaching from the glass and the catalyst regeneration were both demonstrated. A very low leaching ratio (0.03%) was observed after 24 h of treatment, suggesting that deposition of TiO_2_ resulted in good adhesion and stability of the photocatalyst on the surface of borosilicate. This deposition was successfully achieved after calcination of the photocatalyst at 400 °C (TiO_2_-400 °C). The TiO_2_ film was immobilized on glass spheres and the powder was characterized by scanning electron microscopy (SEM), X-ray diffraction and BET. This characterization suggested that thermal treatment did not introduce substantial changes in the measured microstructural characteristics of the photocatalyst. The immobilized photocatalyst degraded more than 96% of the MB in up to 90 min of reaction. The photocatalytic activity decreased after four photocatalytic cycles, but it was recovered by the removal of contaminants adsorbed on the active sites after washing in water under UV-Vis irradiation. Based on these results, the TiO_2_-400 °C coated on glass spheres is potentially a very attractive option for removal of persistent contaminants present in the environment.

## Introduction

Heterogeneous photocatalysis using titanium dioxide (TiO_2_) has been widely investigated as an attractive advanced oxidation process for the physicochemical treatment of a variety of pollutants in water (Malato et al., 2003; Hashimoto, Irie & Fujishima, 2005; Pelaez et al., 2012; Chen et al., 2015; Han et al., 2017; Manassero, Satuf & Alfano, 2017; Nur et al., 2017; Srikanth et al., 2017). TiO_2_ is one of the most used catalysts in photocatalytic degradation of organic pollutants due to properties such as absence of toxicity, very low solubility, high chemical and photostability and low cost (Machado et al., 2012; Wang et al., 2012). Moreover, TiO_2_ absorbs non-negligible portions (3–4%) of available solar energy at the Earth’s surface (Spasiano et al., 2015).

When the absorbed photon energy is equal to or higher than the band gap energy of the semiconductor catalyst, electron–hole pairs are generated. This electron–hole pair has a high and sufficiently positive potential to induce the formation of hydroxyl radicals from water molecules and hydroxyl (OH^−^) ions adsorbed on the semiconductor surface. Hydroxyl radical is the most powerful oxidizing species after fluorine and it can degrade many pollutants due to its low selectivity (Machado et al., 2012; Kim et al., 2016).

The heterogeneous photocatalysis process using TiO_2_ as catalyst in aqueous matrices can be carried out in a slurry reactor or TiO_2_ can be immobilized on various inert support materials, as implemented in fixed-film and fixed-bed reactors (Pozzo, Baltanás & Cassano, 1997; Braham & Harris, 2009; Manassero, Satuf & Alfano, 2017). A slurry reactor is characterized by a high-contact surface area, thus providing greater efficiency due to shorter reaction time and simple operation. This system requires a turbulent regime to ensure that the photocatalyst remain in suspension. However, TiO_2_ removal from the liquid phase after use is a very difficult task. Additionally, the fulfilment of these requirements increases substantially the energy consumption involved in the overall process of the water treatment. In recent years, the use of the photocatalyst supported (immobilized) by different materials, in different reactor configurations has shown to be a good strategy to circumvent these difficulties (Braham & Harris, 2009; Chong et al., 2010; Saleiro et al., 2010; Miranda-García et al., 2010; Spasiano et al., 2015; Amir, Julkapli & Hamid, 2017; Manassero, Satuf & Alfano, 2017; Srikanth et al., 2017).

One of the most widespread commercial catalysts for water/wastewater treatment is the TiO_2_ Aeroxide® P25 (TiO_2_-P25). This catalyst has shown good performance in degrading various persistent organic compounds found in aqueous matrices (Braham & Harris, 2009; Spasiano et al., 2015). The commercial TiO_2_-P25 is a powder mixture of rutile and anatase phases of TiO_2_ with the average particle size ranging from 35 to 65 nm and specific surface area of around 52 m^2^ g^−1^. The better photocatalytic efficiency of this material, as compared to pure rutile and anatase forms of TiO_2_, can be attributed to structural defects caused by the coexistence of anatase (80%) and rutile (20%) phases (Chong et al., 2010; Miranda-García et al., 2010; Pelaez et al., 2012).

In this context, the use of commercial TiO_2_-P25 immobilized on small glass spheres or beads resulted in significant improvement of the photocatalytic treatment. The mix TiO_2_ (sol–gel TiO_2_ and TiO_2_-P25) used for dip-coating immobilization on glass sphere resulted in good treatment performance for degrading emerging contaminants (Miranda-García et al., 2010; Miranda-García et al., 2014) and pesticides (Jiménez et al., 2015) in wastewater using a pilot Compound Parabolic Concentrator (CPC) type reactor. Heat attachment method for the immobilization of TiO_2_-P25 on glass beads was successfully applied to remove dyes (Khataee, 2009; Rasoulifard et al., 2014; Sheidaei & Behnajady, 2015a; Sheidaei & Behnajady, 2015b) and pharmaceuticals (Shankaraiah et al., 2016; Shargh & Behnajady, 2016a; Shargh & Behnajady, 2016b) with the use of UVC radiation instead of sunlight. When two different methods of TiO_2_ immobilization on glass beads (sol–gel and TiO_2_-P25 dispersed in water) were applied to remove Congo red dye, the photocatalytic degradation performed better with the second method (Qiu & Zheng, 2007).

The strength of the TiO_2_ coating adherence to a support is a relevant parameter for immobilized TiO_2_ photocatalysis and this characteristic has been widely investigated by different experimental approaches (Nakata & Fujishima, 2012; Srikanth et al., 2017). Leaching of immobilized TiO_2_ can be also used to assess the adherence strength of TiO_2_ coating. However, to our knowledge, leaching of the TiO_2_ immobilized on glass, so far has not been extensively investigated, which should be done, considering the diversity of immobilized methods that has been applied.

Deactivation and regeneration of the catalyst are relevant aspects to be taken into account when scaling up heterogeneous photocatalysis, due to the economic implications (Miranda-García et al., 2014). Adsorption and strong interaction between the active sites on the catalyst surface and the oxygen-bearing reaction intermediates leads to an abrupt decrease in the number of active sites during catalyst reaction (Prieto, Fermoso & Irusta, 2007). Several types of reactivation methods have been tested to regenerate deactivated photocatalysts, such as: the use of chemicals (HNO_3_, NaOH, NH_4_OH, H_2_O_2_ combined or not with UV irradiation) and water washing (Portela et al., 2007; Kanna et al., 2010; Miranda-García et al., 2014; Yanyan et al., 2017); UV exposure with pure air (Peral & Ollis, 1992); high humidity conditions (Jeong et al., 2013) for air pollution treatment; sonication treatment with water and methanol (Shang et al., 2002) and thermal processes (Cao et al., 2000; Miranda-García et al., 2014; Herrera, Reyes & Colina-Márquez, 2016; Odling et al., 2017).

Based on a recent review on supporting materials for immobilized photocatalytic applications in wastewater treatment (Srikanth et al., 2017), it was concluded that not only new studies focusing on photocatalytic activity under visible and/or solar light spectrum are required, but also recycled over many runs without significant loss in photocatalytic activity. Moreover, this review revealed that more investigations overcoming inherent limitations of immobilized catalysis are required, in order to make future scaling up feasible.

In this context, in the present investigation TiO_2_ was immobilized on glass spheres and evaluated regarding its performance in photocatalytic degradation using methylene blue (MB) as a model of pollutant. MB discoloration was performed in laboratory scale using a CPC type reactor, which has proven to be an efficient and widely applied reactor configuration in photocatalysis experiments (Tanveer & Guyer, 2013), under irradiation simulating the sunlight. A simple approach to catalyst’s immobilization on glass sphere is presented. It employed a suspended nanocrystaline TiO_2_-P25 powder in alcohol and acid medium, with the improved photocatalytic activity by polyethylene glycol (PEG) solution (Miranda-García et al., 2011; Nawawi et al., 2017). The leaching and regeneration capacity of the immobilized TiO_2_ were evaluated in specially designed tests.

## Methodology

### Materials

Titanium dioxide Aeroxide® P25 (TiO_2_-P25) was purchased from Evonik, Brazil. Nitric acid (65%), ethanol and 2-propanol were supplied by Sigma-Aldrich (St. Louis, MO, USA) and Polyethylene glycol PEG-600 (MW: 560–640) by Merck (Darmstadt, Germany). MB was purchased from Cinética (Rio de Janeiro, Brazil). Aqueous solutions containing 10 ppm of MB was prepared with distilled water and used as model of wastewater in the photocatalytic experiments.

### Immobilization of TiO_2_ on glass spheres

Borosilicate glass spheres (Ø = 5 mm) were coated with TiO_2_ film using the dip-coating process. Glass spheres were pre-treated in an ultrasonic bath (Unique, USC-1400A) during 60 min, using a solution of ethanol and distilled water (1:1). After that, the glass spheres were dried at 100 °C for 12 h. The coating of TiO_2_ on glass spheres was done according to methods previously described (Miranda-García et al., 2010; Manassero, Satuf & Alfano, 2017), with few modifications introduced in the present investigation. 6 g of TiO_2_-P25 was added to 150 mL of 2-propanol (being also possible to use ethanol), and the suspension was maintained in ultrasonic bath for 30 min. A volume of 30 µl of nitric acid was added to the suspension, and the obtained material was kept for 30 min in ultrasonic bath. A PEG 600 solution in 2-propanol was then added to reach a final concentration of 200 mg L^−1^, in order to provide a high porosity for the particles (Miranda-García et al., 2010; Miranda-García et al., 2014). This suspension was maintained another 30 min in the ultrasonic bath. After that, the glass spheres were coated by dip-coating with the modified oxide, holding them for 60 s in this suspension, being this process repeated twice. Finally, these glass spheres were dried at 80 °C for 90 min and calcined at 400 °C for 120 min, using a heating rate of 5 °C min^−1^.

In order to know the main properties of the TiO_2_ calcined at 400 °C, a control sample was prepared as follows. The TiO_2_ remained in suspension after the dip-coating process was recovered by solvent removal, using a rotary evaporator (IKA RV10). This material was then submitted to the same thermal treatment applied for the TiO_2_ supported on the glass spheres. The control sample of calcined TiO_2_ is identified hereafter as TiO_2_-400 °C.

### Glass plates to assess titanium leaching in water

Before coating the glass spheres with TiO_2_, a leaching test was done with TiO_2_ supported on glass plates of borosilicate, the same material as the glass spheres. The deposition of TiO_2_-P25 on glass plates were carried out by a similar procedure as described in the previous section. The leaching test of TiO_2_-400 °C on glass plates consisted in the introduction of plates inside a beaker containing ultrapure water (Milliq®; Millipore, Hayward, CA, USA). The plates were kept under water agitation, using a shaker (Quimis, Cascavel, Paraná, Brazil) for 24 h. The amount of titanium leached in water was measured by ICP Optical Emission Spectrometry (OES) (700 series; Agilent Technologies, Santa Clara, CA, USA) in an accredited commercial laboratory (LabAgua, Rio de Janeiro, Brazil), according to the standard method 3120 B (APHA, 2012). All assays were conducted in triplicate.

In order to estimate the mass of the catalyst immobilized on the glass plates, a gravimetric analysis was used. The plates were weighed after moisture removal (110 °C for 1 h), before and after exposure in ultrapure water for 24 h. Then, they were immersed in a 10% solution of nitric acid and maintained under ultrasonic bath for 1 h to remove TiO_2_ film. The plates were then washed and dried (110 °C for 1 h) and weighed again. The leached ratio *(LR)* of Ti was calculated as following (Eq. (1)):

(1) \begin{eqnarray*}LR~(\text{%})= \frac{{W}_{l}}{{W}_{m}} \times 100\end{eqnarray*}

where: *W*_*l*_ is the leached mass of Ti measured by ICP-OES in water, and *W*_*m*_ is the initial mass of TiO_2_ immobilized on glass plates.

### Materials characterization

The powder samples of TiO_2_-P25 and TiO_2_-400 °C were characterized by X-ray powder diffraction (XRD) and Brunauer–Emmett–Teller (BET) N_2_ adsorption. In addition, TiO_2_ immobilized on glass spheres images were collected using scanning electron microscopy (SEM). These techniques are briefly described below.

### XRD

X-ray powder diffraction (XRD) patterns of the samples were obtained with a Brüker D8 Focus diffractometer in the Bragg-Brentano geometry, using Cu *K*-alpha radiation and a secondary graphite crystal monochromator. The diffraction patterns were collected over a 2Θ range of 10°–80° at a step of 0.02° 2Θ and acquisition time of 20 s per step with scintillation detector. The phase composition determination and the estimation of mean crystallite size were performed employing the Topas-Academic software. Instrument function was obtained at the same instrumental configuration using NIST standard reference material SRM1976 (*α*-Al_2_O_3_).

The Rietveld method of powder diffraction pattern fitting and structural (microstructural) refinement by convolution approach to peaks profile modeling implemented in the Topas-Academic software was used to obtain the crystalline phase composition and the average crystallite sizes of the respective phases. The Voigt function was employed to model the crystallites size effect, which consists in a characteristic broadening of X-ray diffraction peaks. Isotropic model of size broadening which implies a spherical shape of crystallites well described the diffraction peak profiles. A sample average volume weighted thicknesses of crystallites defined by the Stokes’ and Wilson’s equation (Stokes & Wilson, 1942) were calculated using Eq. (2):

(2) \begin{eqnarray*}{L}_{V}= \frac{\lambda }{\beta \,\cos \nolimits (\theta )} \end{eqnarray*}

where: *β* is the integral breadth of the diffraction line (peak area divided by peak maximum), *λ* is the wavelength of the X-rays and *θ* is the half of the diffraction angle.

### SEM

The TiO_2_ coated glass spheres were examined by scanning electron microscopy (SEM) in order to characterize the surface morphology of the TiO_2_ films and aggregates, including the eventual formation of large-sized TiO_2_ agglomerations that can lead to cracks formation, which can lead to detrimental effects on long-term mechanical stability of the coating in applications involving water matrix. SEM images of TiO_2_-coated glass spheres, before and after photocatalysis experiments were acquired using a Helios Nanolab 650 Dual Beam. The SEM images were obtained using 2 kV, 13 pA, FEG filament and using ETD and TLD detectors. Samples were dropped in carbon conductive adhesive tapes.

### BET

For the determination measurements of the specific surface area and total pore volume, Brunauer–Emmett–Teller (BET) N_2_ adsorption were carried out using a Autosorb-1 analyzer (Quantachrome Instruments, Boynton Beach, FL, USA) automated apparatus using liquid N_2_ adsorption at a temperature of 140 °C. Specific surface area was determined in the relative pressure range between 0.05 and 0.3.

### Photodegradation tests

#### CPC reactor

**Figure 1:**
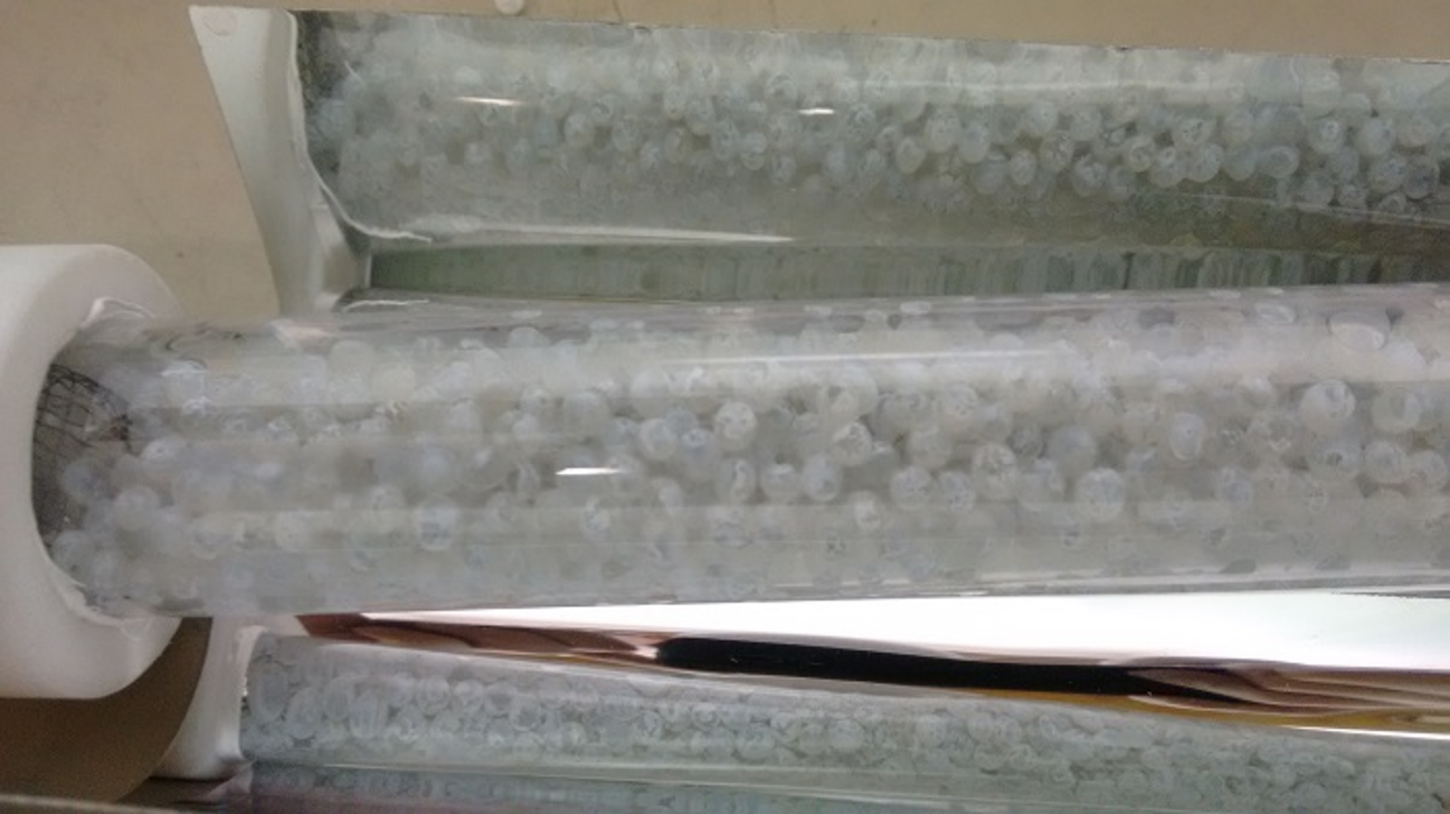
Glass spheres coated with TiO_2_-400 °C, filling borosilicate tubes in the CPC reactor.

The evaluation of the photocatalytic and adsorption capability of the TiO_2_-400 °C catalyst immobilized on glass spheres was done with a lab scale compound parabolic collector reactor (CPC), using an Ultra-vitaluz OSRAM 300W lamp, that simulates the solar spectrum (Heredia, Sham & Farfán-Torres, 2015). The average irradiance was adjusted around 30 Wm^−2^ of UVA-light intensity, using a Delta Ohm model HD-2302 radiometer. The reactor was built using a borosilicate glass tube (external diameter of 30 mm, wall thickness of 2.0 mm, and length of 200 mm) and a polished anodized aluminum surface, made in the form of an involute, used as reflector (Fig. 1) (Rodríguez et al., 2004; Duarte et al., 2005). The schematic drawing of photodegradation experiments is presented in Fig. S1, Appendix S1.

The flow rate used in all experiments was 500 mL min^−1^. A peristaltic pump (Watson-Marlon 502S) and a magnetic stirrer were used to recirculate and homogenize the 500 mL of the 10 ppm MB-containing solution. The temperature and pH of the solution varied, respectively, from 25 °C to 30 °C and from 6.3 to 6.7 throughout the experiment. Samples of the solution (4 mL) were collected during the experiment, being the absorbance measured at 664 nm using a HACH DR 5000 UV-Vis spectrophotometer. Five treatment cycles were applied with the same photocatalyst. The solution was recirculated under dark condition for 30 min to ensure adsorption–desorption equilibrium before illumination (Miranda-García et al., 2010; Zhang et al., 2015), only for the first cycle. To regenerate the catalyst, before the fifth cycle, distilled water was recirculated, under irradiation, into the reactor during 180 min. The efficiency of MB removal solution was calculated according to Eq. (3):

(3) \begin{eqnarray*}E=(1-Cn/Co)\times 100\end{eqnarray*}

where: *Cn* is the MB concentration at a time *t* from the beginning of the recirculation test and *Co* is the initial MB concentration.

The pseudo first-order apparent rate constant (*k*, min^−1^) related to the discoloration of MB, an approximate measure of the photocatalytic activity (Borges et al., 2016) was determined from the regression curve ln*(Cn/Co) vs.* irradiation time (França et al., 2016).

## Results and Discussion

### Materials characterization

**Figure 2:**
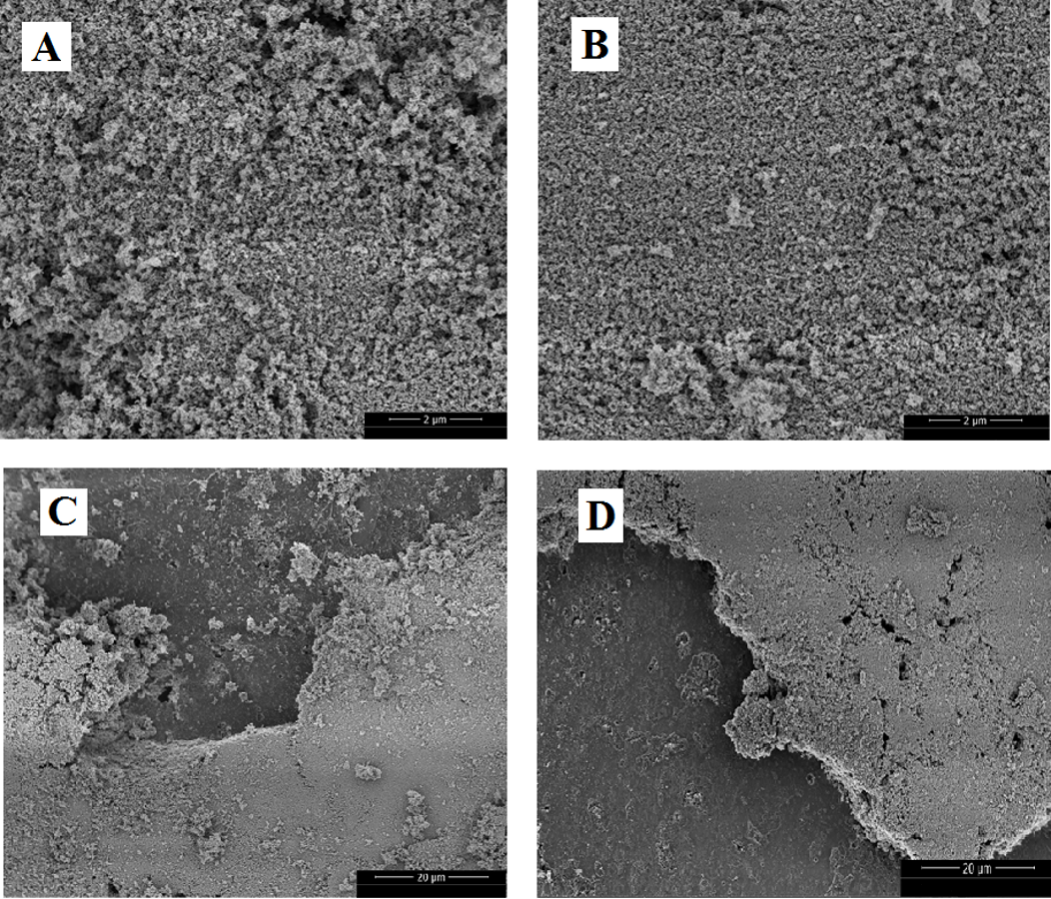
SEM images of TiO_2_-400 °C on glass spheres surface before use (A and C) and after five photocatalytic treatment cycles (B and D).

Figure 2 shows the selected SEM images of the surface of the catalyst on glass spheres, before the photocatalytic treatment (*A* and *C*) and after five cycles of treatment (*B* and *D*).

The rough surface morphology of TiO_2_-400 °C films and presence of agglomerates composed of TiO_2_ nanoparticles, observed in the present study seems to be similar to the reported by other researchers (Chen & Dionysiou, 2006; Miranda-García et al., 2010; Khalilian et al., 2015). The SEM images (Fig. 2) also confirm that the morphology of the immobilized TiO_2_-400 °C remains mostly unchanged after several cycles of photocatalytic experiments, suggesting that the deposition of TiO_2_ over the glass spheres was efficient and a stable support was achieved.

### Micro-structural characterization of TiO_2_ samples

**Figure 3:**
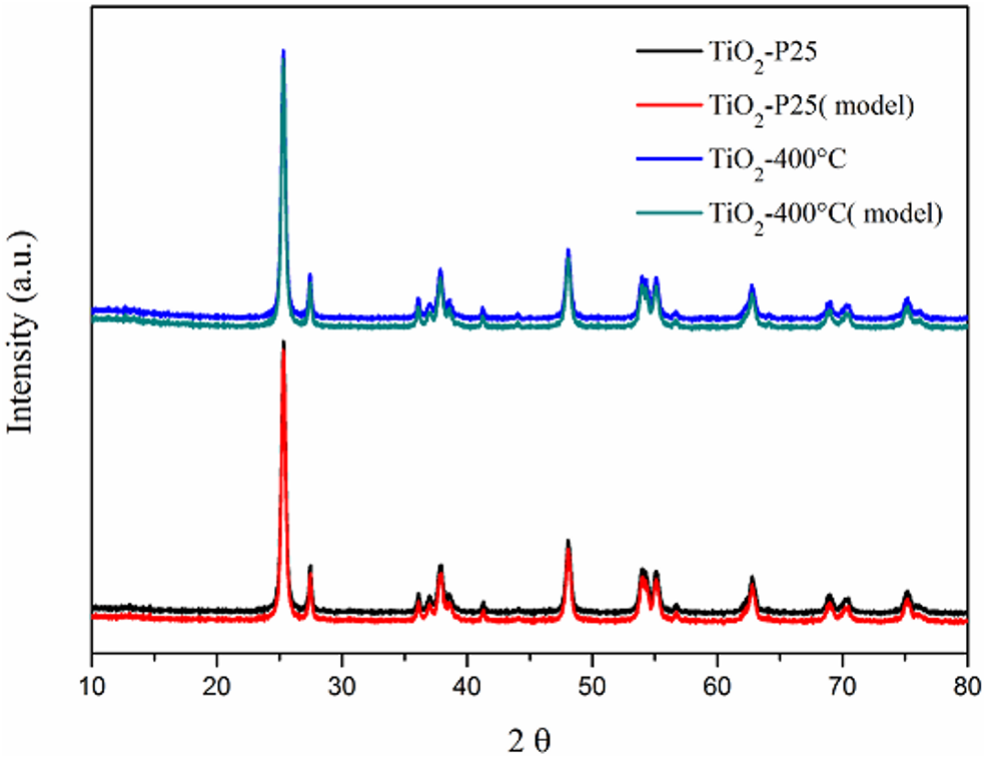
X-ray diffraction patterns of TiO_2_-P25 and TiO_2_-400 °C.

The XRD patterns of the TiO_2_-P25 and TiO_2_-400 °C nanopowders are shown in Fig. 3. As expected, a two-phase material composed of anatase and rutile is perfectly in accordance with the standards observed in the X-ray diffraction samples. The main microstructural parameters of both samples, calculated from Rietveld refinement (crystalline phase composition and crystallite size, *Lv*) are shown in Table 1.

**Figure 4:**
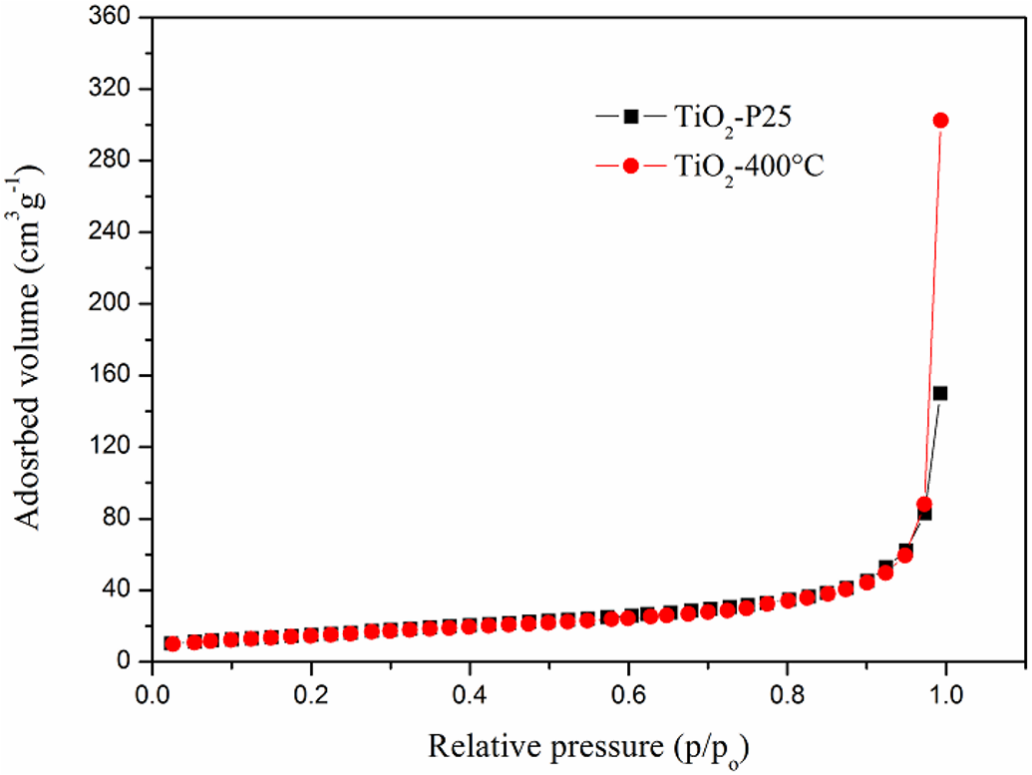
Nitrogen adsorption isotherms of the TiO_2_-P25 powder before and after calcination at 400 °C (TiO_2_-400 °C).

As one can see from Table 1, the thermal treatment of TiO_2_-P25 did not produce any substantial changes in the measured microstructural characteristics. Both, phase composition and average crystallite size, remained unaltered within the statistical errors of data modelling. This is confirmed by BET measurements. Figure 4 shows the nitrogen adsorption isotherms for TiO_2_-P25 and TiO_2_-400 °C. Type II adsorption isotherms were obtained, indicating a macroporous nature of the adsorbent with strong adsorbate-adsorbent interactions (Buddee et al., 2014).

**Table 1:** Microstructural properties of TiO_2_-P25 and TiO_2_-400 °C.

**Material**	Phase content (%)		Crystalline size, *Lv* (nm)		*S*_BET_	Pore volume
	Anatase	Rutile	Anatase	Rutile	(m^2^ g^−1^)	(cm^3^ g^−1^)
TiO_2_-P25	86(1)	14(1)	20.5(8)	31(6)	56.2	0.129
TiO_2_-400 °C	87(1)	13(1)	21.1(8)	30(6)	53.9	0.137

The specific surface area and the total pore volume of TiO_2_-P25, Table 1 do not change significantly after calcination. Nevertheless, a small reduction of the specific surface area and a small increase in the pore volume can be indicative of the formation of slightly larger or/and more regularly shaped (more spherical) TiO_2_ particles. This trend in the behaviour of the above mentioned properties of nanocrystaline TiO_2_ powders, due to an increase in the calcination temperature, has been reported in previous studies (Chen & Dionysiou, 2007; Viswanathan & Raj, 2009; Wang et al., 2012). These changes can be attributed to defects induced by the temperature annealing and coalescence of the crystallites. However, the character and the extent of the overall changes in the microstructural features corroborate with a limitation in the annealing processes in near-surface regions of the TiO_2_ particles. Consequently, the expected photocatalytic activity of the TiO_2_-400 °C immobilized on glass spheres is very similar to that of TiO_2_-P25.

### Adherence of the TiO_2_ catalyst on glass plates

The adherence strength of the TiO_2_-400 °C immobilized on glass plates was evaluated by exposing the photocatalyst coating to stirring in ultrapure water (Milliq) for 24 h and subsequent measurement of the amount of TiO_2_ leached into the water. The assay was conducted in triplicates together with negative control samples (a blank using a glass plate). No visually detectable depletion of TiO_2_-400 °C coating on glass plates was observed after the treatment. The measured average TiO_2_ concentration in water after stirring the TiO_2_-400 °C coated on glass plates was 2.7 (±1.0) µg L^−1^. No TiO_2_ was detected in the water after stirring the negative control plate. The catalyst mass deposited on each plate was estimated as being equal to 16.6 (±1.9) mg L^−1^. The LR value was obtained to be 0.03% of TiO_2_ leached in water after 24 h. This LR value can be considered small when compared with data reported in other studies. For example, the LR of the TiO_2_ immobilized on glass plates was found to be (1.52 ± 0.12%) after 10 h of stirring treatment (Nawi et al., 2011), while in a recent study (Lam et al., 2017) approximately 10% of the TiO_2_ immobilized on glass beads was leached into water. In addition it was reported (Jawad et al., 2016) that 7.3% of the TiO_2_ of a TiO_2_/epoxidized natural rubber (ENR) immobilized on glass plates was leached after 4 h.

### Methylene Blue (MB) degradation

To confirm the photocatalytic activity of the TiO_2_-400 °C immobilized on glass spheres as produced in the present investigation, the degradation of MB, used as a model of oxidizable substrate, expressed in terms of its discoloration, was evaluated.

At the beginning, the glass spheres were inserted in the reactor and washed several times with distilled water to eliminate non-bonded or weakly bonded TiO_2_ and to avoid any effect produced by suspended TiO_2_ nanoparticles, eventually released from the immobilized photocatalyst.

In a typical experimental cycle, around 1,500 spheres (Ø = 5 mm) coated by the photocatalyst (around 0.3 ± 0.1 mg of TiO_2_ immobilized per sphere) were placed into the glass tube of the CPC reactor. The thickness of the TiO_2_-400 °C layer on glass spheres was estimated as a function of the mass in each sphere (about 0.3 mg), density and total layer volume (glass sphere volume plus TiO_2_ layer). For calculation purpose, the TiO_2_ layer considered uniform and a cubic equation was used. The value obtained was about 1 µm. This value was considered reasonable in comparison to previous studies (Negishi et al., 2007; Espino-Estévez et al., 2015).

**Figure 5:**
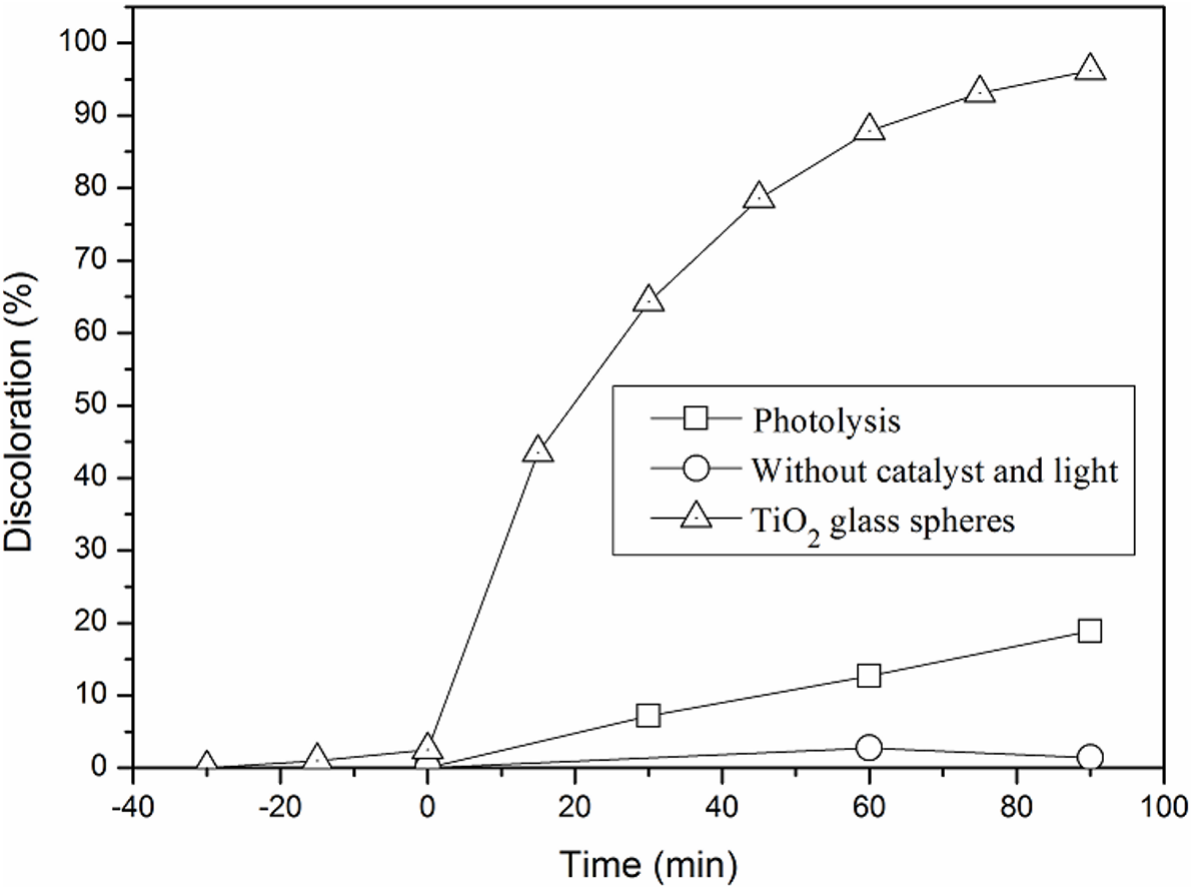
Methylene blue (MB) adsorption in the CPC reactor (without catalyst and light) and its degradation (measured as discoloration %) by photolysis and photocatalytic using glass spheres coated by TiO_2_-400 °C.

As reported before, the MB degradation was carried out for 90 min using an artificial lamp that simulates the solar spectrum in the CPC reactor. Before the photocatalytic experiments, the level of adsorption of MB on the photocatalyst was evaluated in experiments involving the recirculation of the solution in the absence of light, and the MB degradation by photolysis was assessed, using the same lamp as irradiation font. In order to evaluate other possible MB adsorption processes in the CPC reactor, the solution containing MB was recirculated under similar experimental conditions, but without the presence of catalyst and light. Figure 5 shows the results of the MB degradation mediated by TiO_2_-400 °C supported on glass spheres, together with the non-catalytic photolysis and MB adsorption (without catalyst nor irradiation), carried out in the CPC system.

As shows in Fig. 5, in the first cycle with TiO_2_ glass spheres the colour given by MB was almost completely removed (96%) in 90 min of photocatalytic reaction and that only 19% of the initial colour disappeared due to photolysis. It was also observed that the adsorption of MB in the catalyst is minimal around the rated range. This result confirms those obtained in previous investigations (Munjal, Dwivedi & Bhaskarwar, 2015; Cha et al., 2017). For this reason, the adsorption of the immobilized material during subsequent cycles was considered negligible.

**Figure 6:**
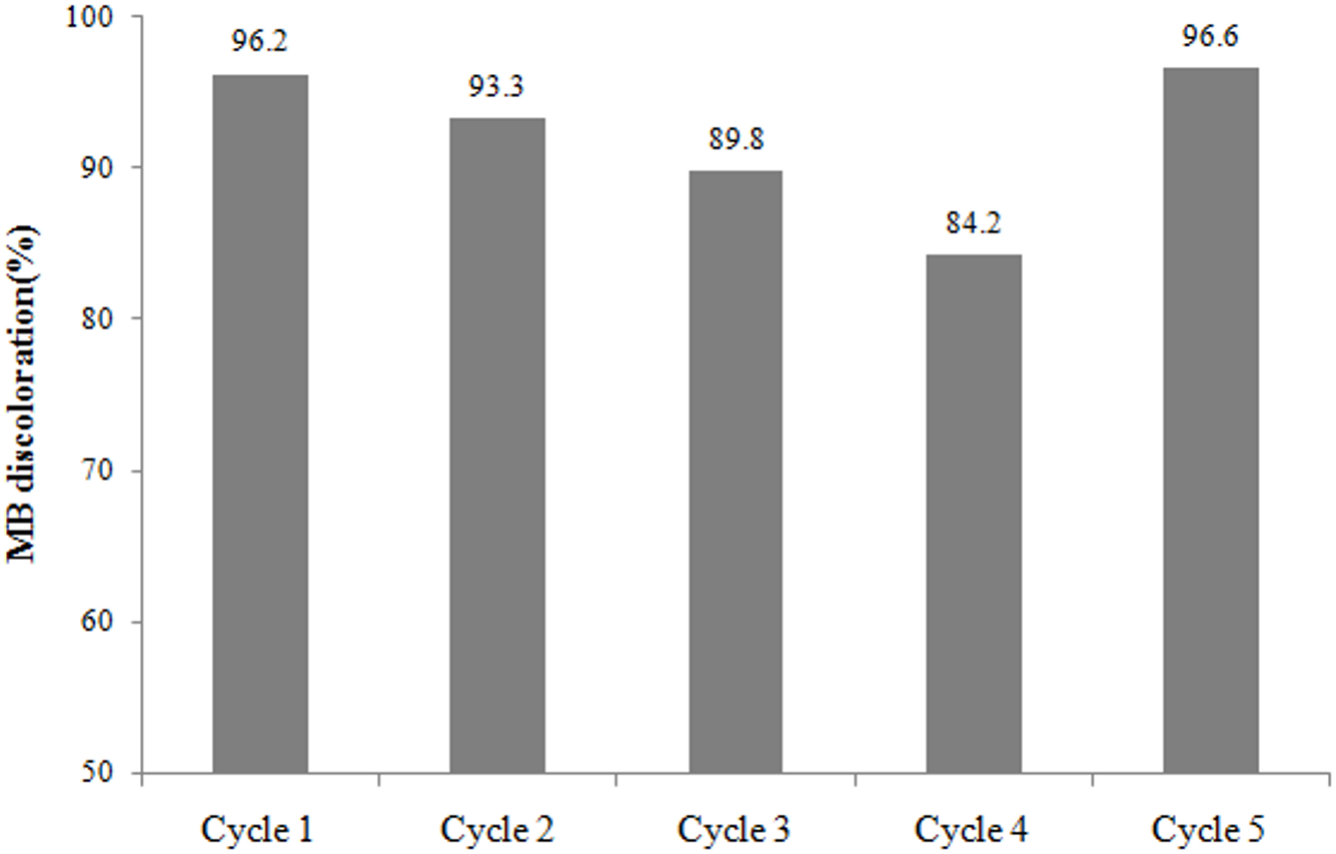
Evolution of methylene blue (MB) discoloration (%) with the number of cycles.

The remaining photocatalytic capacity of used material was checked, following a series of five photocatalytic cycles. Before running the last cycle, distilled water was recirculated into the CPC reactor containing the glass spheres coated with TiO_2_-400 °C, under irradiation during 180 min. The levels of discoloration after 90 min of reaction, in terms of percentage and their respective pseudo-first order kinetics constants are shown, respectively, in Fig. 6 and Table 2.

**Table 2:** Pseudo first order kinetics constant *k* (min^−1^) for MB discoloration in each cycle (±standard error).

	Cycle 1	Cycle 2	Cycle 3	Cycle 4	Cycle 5
*k*	0.036 (0.00062)	0.030 (0.00028)	0.025 (0.00028)	0.020 (0.00005)	0.039 (0.002)

As shown in Fig. 6, the degradation efficiency using TiO_2_-400 °C supported on glass spheres suffered a gradual decrease with repeated use. The photocatalytic activity was recovered after the fourth cycle with the washing of the catalyst under solar radiation simulated for 180 min, which can be viewed on the fifth cycle of degradation. The observed deactivation of the catalyst should be due to strong interactions between the active sites on the catalyst surface and the dye, as well as to the dye adsorption, leading to loss of function of part of the active sites of the catalyst. The blue color characteristic of MB found in the glass spheres containing the catalyst observed after each cycle of the photocatalytic treatment supports this hypothesis. A similar effect has been reported previously (Kanna et al., 2010; Salehi, Hashemipour & Mirzaee, 2012).

The pseudo first order kinetics constants (Table 2) corroborates the statements above, since the kinetics constants decreases in sequence after each cycle of treatment, unless for the last cycle, which used the catalyst after its regeneration.

In a recent investigation, after 180 min of irradiation about 10 and 90% of the MB in solution (10 mg L^−1^) was degraded using respectively, TiO_2_ and N-doped TiO_2_ immobilized on glass bead (Kassahun et al., 2017). Compared to those results, the immobilized TiO_2_-400 °C photocatalyst used in the present investigation represents a breakthrough in the knowledge, since in our study it was possible to degrade in 90 min more than 90% of the initial concentration of the same dye (MB). Moreover, the present investigation reached an effective regeneration of photocatalytic activity applied to MB removal, using only distillate water and simulated solar radiation by lamp (UV-vis light), which is a simple and non-expensive approach to efficiency recovery of material.

## Conclusions

The present investigation describes a simple procedure to obtain an efficient TiO_2_-based photocatalyst immobilized through a very stable coating and with good adhesion on borosilicate glass spheres. SEM microscopy, XRD and BET showed that microstructural features of TiO_2_-P25 remained almost unaltered after calcination at 400 °C. Generally, the main limitation of immobilization of photocatalysts is not only in the loss of photocatalytic activity, but also due the decrease of the coating integrity during its use. This is not the case of the material presented in this study, since the photocatalyst immobilization did not compromise its activity, and the adhesion on glass spheres proved to be quite effective even after exhaustive leaching tests in water.

Under simulated solar irradiation at conservative power (30 W m^−2^ within the UVA range), using a lab scale CPC reactor, the immobilized TiO_2_-400 °C photocatalyst could degrade up to 96% of the MB present in aqueous solutions in 90 min of reaction. The reuse of the photocatalyst resulted in a decrease of its photocatalytic activity due to the adsorption of MB in active sites of the catalyst, which could be reversed by washing with water, under simulated solar irradiation for 180 min. This result confirms the effective regeneration of the photocatalyst by a simple and non-expensive approach. In addition, the immobilized TiO_2_-400 °C on glass spheres obtained through the procedure described in the present study is a promising indication of its applicability to environmental remediation and wastewater/water treatment based on heterogeneous photocatalysis.

##  Supplemental Information

10.7717/peerj.4464/supp-1 Appendix S1 Schematic drawing of photodegradation experiments (1) beaker; (2) magnetic stirrer; (3) peristaltic pump; (4) CPC reactor (collector and borosilicate glass tube); and (5) solar spectrum simulated lamp.

10.7717/peerj.4464/supp-2 Data S1 Raw data

## Additional Information and Declarations

### Data Availability

The following information was supplied regarding data availability: The raw data are provided in Data S1.
