# Supplementary figures and images for: Immobilized TiO2 on glass spheres applied to heterogeneous photocatalysis: photoactivity, leaching and regeneration process
Source: PeerJ. 2018 Mar 6;6:e4464. doi: 10.7717/peerj.4464 (PMC5844248; doi:10.7717/peerj.4464)

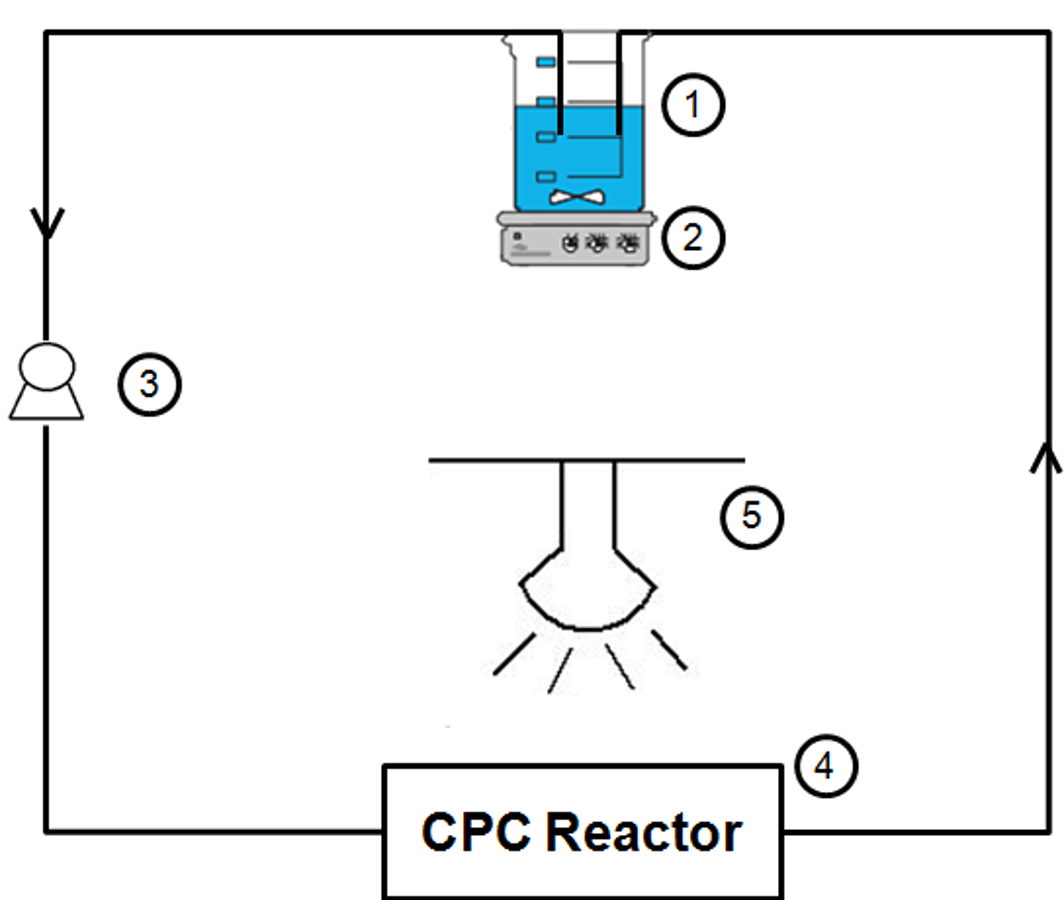

Supplement: Appendix S1 — (1) beaker; (2) magnetic stirrer; (3) peristaltic pump; (4) CPC reactor (collector and borosilicate glass tube); and (5) solar spectrum simulated lamp. [file peerj-06-4464-s001.png]
